# A Novel *N-Tert*-Butyl Derivatives of Pseudothiohydantoin as Potential Target in Anti-Cancer Therapy

**DOI:** 10.3390/molecules26092612

**Published:** 2021-04-29

**Authors:** Daria Kupczyk, Renata Studzińska, Szymon Baumgart, Rafał Bilski, Tomasz Kosmalski, Renata Kołodziejska, Alina Woźniak

**Affiliations:** 1 Department of Medical Biology and Biochemistry, Faculty of Medicine, Collegium Medicum in Bydgoszcz, Nicolaus Copernicus University in Toruń, Karłowicza 24, 85–092 Bydgoszcz, Poland; rafal.bilski@cm.umk.pl (R.B.); renatak@cm.umk.pl (R.K.); alina-wozniak@wp.pl (A.W.); 2 Department of Organic Chemistry, Faculty of Pharmacy, Collegium Medicum in Bydgoszcz, Nicolaus Copernicus University in Toruń, Jurasza 2, 85–089 Bydgoszcz, Poland; sz.baumgart@cm.umk.pl (S.B.); tkosm@cm.umk.pl (T.K.)

**Keywords:** carcinogenesis, anti-cancer therapy, glucocorticoids, thiazolone derivatives, enzyme inhibition, 11β-hydroxysteroid dehydrogenase, cell proliferation

## Abstract

Tumors are currently more and more common all over the world; hence, attempts are being made to explain the biochemical processes underlying their development. The search for new therapeutic pathways, with particular emphasis on enzymatic activity and its modulation regulating the level of glucocorticosteroids, may contribute to the development and implementation of new therapeutic options in the treatment process. Our research focuses on understanding the role of 11β-HSD1 and 11β-HSD2 as factors involved in the differentiation and proliferation of neoplastic cells. In this work, we obtained the 9 novel *N*-*tert*-butyl substituted 2-aminothiazol-4(5*H*)-one (pseudothiohydantoin) derivatives, differing in the substituents at C-5 of the thiazole ring. The inhibitory activity and selectivity of the obtained derivatives in relation to two isoforms of 11β-HSD were evaluated. The highest inhibitory activity for 11β-HSD1 showed compound **3h**, containing the cyclohexane substituent at the 5-position of the thiazole ring in the *spiro* system (82.5% at a conc. 10 µM). On the other hand, the derivative **3f** with the phenyl substituent at C-5 showed the highest inhibition of 11β-HSD2 (53.57% at a conc. of 10 µM). A low selectivity in the inhibition of 11β-HSD2 was observed but, unlike 18β-glycyrrhetinic acid, these compounds were found to inhibit the activity of 11β-HSD2 to a greater extent than 11β-HSD1, which makes them attractive for further research on their anti-cancer activity.

## 1. Introduction

Cancer is an important public health threat, becoming an increasingly widespread disease worldwide. Despite years of research, the biochemical processes underlying the phenomenon of neoplasms are still not fully understood [1]. Particular attention is paid to the interaction between glucocorticoids, inflammation and the neoplastic process. A representative example of a glucocorticoid is cortisol, which is a hormone produced by the cortex of the adrenal glands. Both acute and chronic stress, which stimulates the adrenal cortex to secrete glucocorticosteroids, can lead to changes in its daily secretion. These, in turn, affect the metabolism of proteins, carbohydrates and fats as well as the course of inflammatory processes [2,3]. Adequate modulation of the level of glucorticosteroids is necessary for the proper functioning of the body. 

**Scheme 1:**
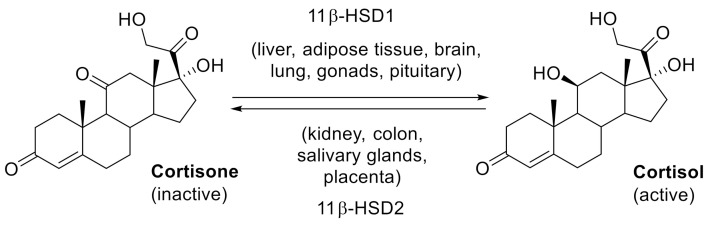
The physiological role of the two isoforms of 11β-HSD.

Type 1 11β-hydroxysteroid dehydrogenase (11β-HSD1) and type 2 (11β-HSD2) play an important role in the local inactivation of glucocorticosteroids. In addition, they catalyze the interconversion of active glucorticoids to their inactive counterparts, such as the conversion of cortisol to cortisone by 11β-HSD2 and a reverse reaction catalyzed by 11β-HSD1 leading to the formation of active cortisol (Scheme 1).

The immune system plays an extremely important role during the disease process, including the formation of cancer cells, for example, macrophages, show the promoting effects on tumors through many different mechanisms, including promotion of tumor cell invasion and metastasis formation, stimulation of angiogenesis, and immunosuppressive effects [4]. The studies showed a significant correlation between the level of NK cells produced and the expression of 11β-HSD1. In the tissue which had ≤5% NK cells, the level of 11β-HSD1 was significantly higher (approximately 45% of immupositive cells) while in the tissue with >5% NK cells the level of 11β-HSD1 was significantly lower (approximately 15% of immunopositive cells). Effective inhibition of this enzyme contributed to an increase in the number of NK cells, and thus a stronger response on the part of the immune system. 11β-HSD2 has been shown to play a significant role in the formation and growth of neoplasms, in particular colon and lung cancer. Studies showed increased expression of 11β-HSD2 in cancer cells in comparison to normal cells in mice. Moreover, due to its physiological function, this isoform is also an active inhibitor of gluconeogenesis, a process dependent on the level of glucocorticoids. 11β-HSD2 promotes the proliferation of neoplastic liver cells due to cortisol inactivation, which leads to reduced inflammatory response in the liver. In pituary tumor cells, 11β-HSD1 expression was significantly lower (0.2-fold), while 11β-HSD2 expression was higher (10-fold) in comparison with regular cells. Therefore, it is important to define the role of 11β-HSD1 and 2 in the regulation of glucocorticoid levels during cancer development and progression. The above-mentioned enzymes can modulate glucocorticoid activity in a strictly defined and tissue-specific way [5,6,7,8]. Hence, an important issue is the influence on the regulation of the activity of these enzymes. It seems particularly important to learn about effective compounds that are selective inhibitors of these enzymes. Understanding the role of 11β-HSD inhibitors in the development and progression of individual types of cancer may have a potentially significant impact on the therapy and further cancer development. For example, in non-melanoma skin cancers, the participation of 11β-HSD isoenzymes seems to be a potential therapeutic target due to the regulation of both activation and deactivation of glucocorticosteroids, which function at the level of pre-receptors. The studies showed abnormal 11β-HSD2 expression in cancer cells as compared to non-neoplastic cells, which suggests the role of 11β-HSD2 in neoplastic transformation. Consequently, it also indicates the possibility of using 11β-HSD2 inhibitors as a potential therapeutic target [9]. In turn, Chang et al. demonstrated in lung tumors that the participation of this enzyme is correlated with greater expression of tumor cells and, at the same time, found that inhibition of 11β-HSD2 activity contributed to a significant slowdown in tumor growth and development [10]. In animal models, inhibition of 11β-HSD2 was also observed to prevent adenocarcinoma, tumor growth and metastasis [11]. The increase in 11β-HSD2 expression inhibits the secretion of cortisol, a hormone with an antiproliferative effect. In the studies of Koyama et al. and Lipka et al. concerning breast cancer, it has been proven that overexpression of 11β-HSD2 in some cells leads to their uncontrolled growth. Therefore, it can be concluded that the inhibition of this enzyme is the basis for limiting the growth of cancer cells in breast cancer [12,13]. In the case of prostate cancer, studies have shown an increased activity of 11β-HSD2 in neoplastic cells. Moreover, the disturbance in the activity of 11β-HSD isoenzymes is important in the progression of neoplasms with impaired androgen secretion [14]. 

Glucocorticoids are known for their antiproliferative function. Hence, the regulation of their secretion is crucial for the maintenance of normal cellular processes within the human body. An imbalance in their secretion may promote the formation of cancer cells or tumors and their spread in the process of metastasis [6]. In view of these facts, it seems clear that an attempt to find tissue-specific inhibitors of 11β-HSD2 will allow the development of optimal therapeutic strategies.

**Scheme 2:**
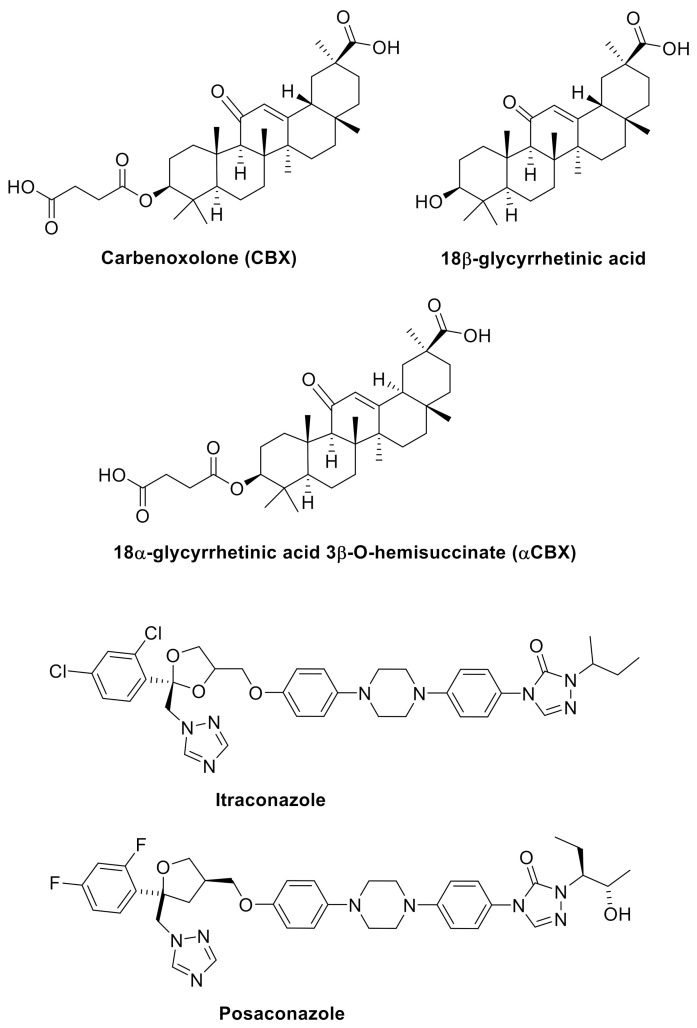
Carbenoxolone and the inhibitors of 11β-HSD2.

A known substance that inhibits the activity of 11β-HSD2 is glycyrrhetinic acid (18β-glycyrrhetinic acid, 18β-GA). However, it is a non-selective inhibitor as it also significantly inhibits the enzyme isoform 1 activity [15,16]. An effective inhibitor is αCBX (18α-glycyrrhetinic acid 3β-O-hemisuccinate, αCBX), which is a diastereoisomer of carbenoxolone (CBX), a known inhibitor of the 11β-HSD isoform [17]. Among the non-steroidal inhibitors, fungicides should be mentioned: itraconazole and posaconazole, which are derivatives of triazole (Scheme 2) [18]. However, due to the fact that studies on the practical use of these compounds to inhibit the activity of 11β-HSD2 have not been conducted, it is justified to search for new selective inhibitors that could be used as a potential therapeutic target in anti-cancer therapy.

While searching for selective inhibitors of the 11β-HSD isoform, our attention was focused on compounds containing the thiazole ring [19,20,21]. Some 2-aminothiazol-4(5*H*)-one derivatives have been shown to be selective inhibitors of 11β-HSD1. During these studies, we became interested in those compounds that did not inhibit the activity of isoform 1, but were selective 11β-HSD2 inhibitors [22]. Encouraged by the obtained results, we decided to synthesize another series of derivatives containing a *tert*-butyl substituent in the amino group and to evaluate their usefulness in inhibiting the activity of both 11β-HSD isoforms.

## 2. Results and Discussion

### 2.1. Chemistry and in Vitro Studies

**Scheme 3:**
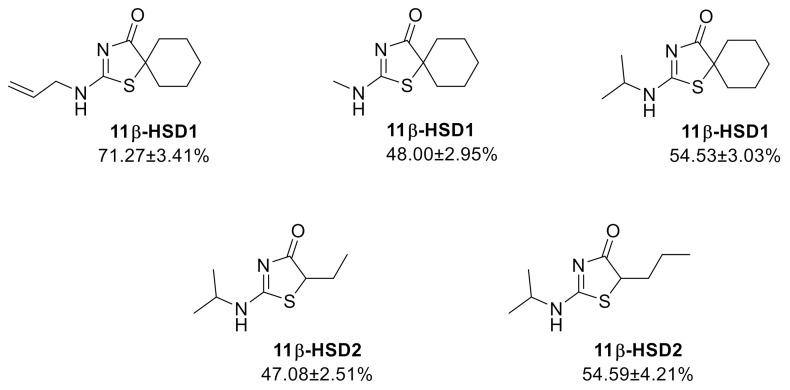
Previously synthesized inhibitors of 11β-HSD1 and 11β-HSD2.

The studies carried out for the previously obtained 2-aminothiazol-4(5*H*)-one derivatives have shown that the inhibitory activity in relation to both 11β-HSD1 and 11β-HSD2 is influenced by the type of substituent at C^5^ as well as the moiety attached to the amino group at C-2 [20,21,22]. Regardless of the type of the substituent in the amino group, the highest inhibitory activity in relation to 11β-HSD1 was demonstrated by compounds containing the *spiro* thiazole and cyclohexane ring system. In relation to 11β-HSD2, the most active compounds have been found to contain simple alkyl substituents in the 5-position and an isopropyl substituent at the amino group (Scheme 3).

The analysis of the results obtained in conjunction with the molecular modeling carried out for a series of compounds with an allyl and methyl substituent allowed to conclude that a larger hydrophobic substituent in the amino group should increase the interaction of potential inhibitors with the enzyme’s active center [20,21]. Therefore, our attention has been focused on 2-*tert*-butylaminothiazol-4(5*H*)-one derivatives.

**Scheme 4:**
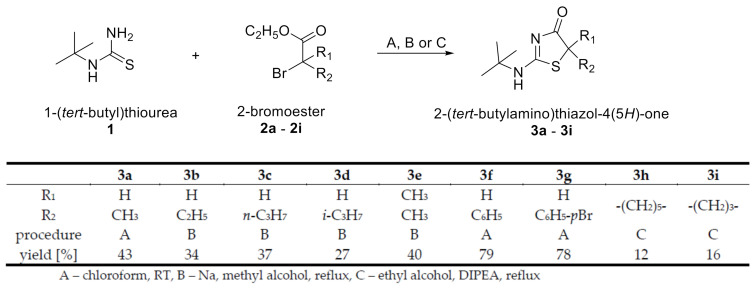
Synthesis of 2-(*tert*-butylamino)thiazol-4(5*H*)-one deriatives.

Series of 2-(*tert*-butylamino)thiazol-4(5*H*)-one derivatives was synthetized by reaction of 1-(*tert*-butyl)thiourea with appropriate 2-bromoesters, with different substituents on the nitrogen atom (Scheme 4). 

**Scheme 5:**
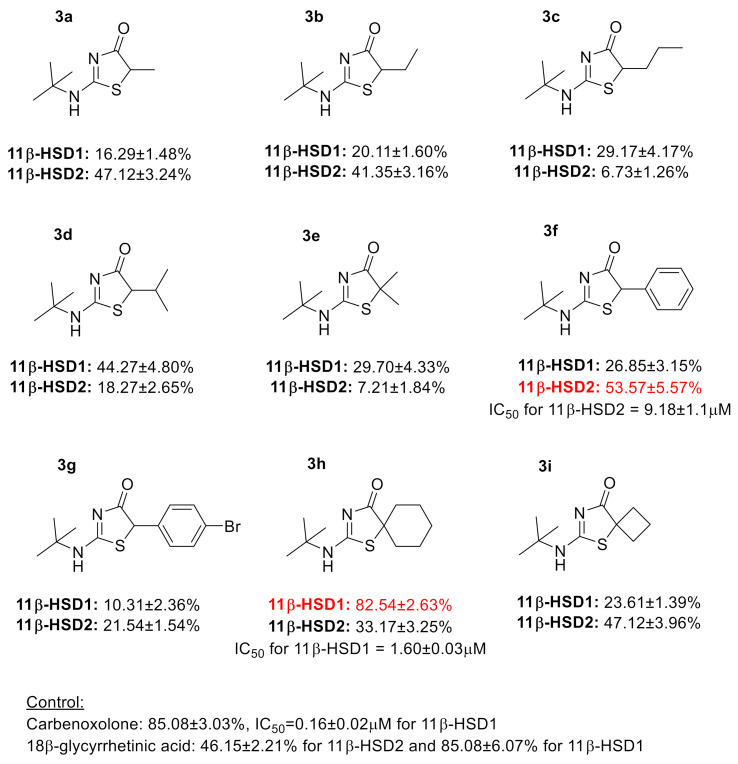
Inhibitory activity of 2-(*tert*-butylamino)thiazol-4(5*H*)-one deriatives.

The derivatives obtained all have a *tert*-butylamino substituent in the 2-position, but differ in the 5-position of the thiazole ring. The obtained compounds were tested in vitro for the inhibition of 11β-HSD1 and 11β-HSD2 in order to assess their selective action and thus their usefulness as potential therapeutic agents that could effectively support the treatment of neoplastic disease. Further advanced research could influence the understanding of the pathophysiology of these diseases, and thus the use of appropriate 11β-HSD inhibitors in the therapeutic process. The above data justify further research in order to provide detailed information on the role and use of potential 11β-HSD inhibitors in the cancer process. (Scheme 5).

Among 9 synthesized new derivatives, the compound **3h** showed the highest activity towards the inhibition of 11β-HSD1. It has already been shown that the presence of the *spiro* system of the thiazole and cyclohexane rings has a large influence on the location of the molecule in the active center of the enzyme, and thus on its high activity compared to derivatives containing other substituents in the 5-position of the thiazole ring [20,21]. Additionally, the increase in the volume of the substituent in the amino group (introduction of the *tert*-butyl substituent) contributed to the increased activity in comparison with derivatives with a methyl, isopropyl or allyl substituent.

The introduction of the *tert*-butyl group to the molecule resulted in a significant increase in the inhibitory activity towards 11β-HSD2 compared to the isopropyl group [22] for derivatives having a methyl and phenyl substituent at C-5 (compounds **3a** and **3f**) and a decrease for the derivative with an ethyl group (**3b**). However, a decrease in selectivity in the inhibition of 11β-HSD2 is observed compared to isopropyl-containing derivatives. It is worth noting that, unlike 18β-glycyrrhetinic acid, these compounds, as well as isopropyl derivatives, inhibit the activity of 11β-HSD2 to a greater extent than 11β-HSD1. These results allow us to outline future prospects for using these inhibitors as potential drugs for use in treatment of neoplastic diseases. A small amount of data from the literature prompts our team to expand and continue research in this area.

Research by Cirillo et al. showed that both the activity of 11β-HSD1 and 11β-HSD2 in malignant skin tumors was dysregulated. The use of an inhibitor in the form of glyceritic acid led to inhibition of 11β-HSD2 expression, contributing to an increase in cortisol levels, thus ensuring antiproliferative activity [23]. In turn, the research of Jiang et al. demonstrated the therapeutic potential of 11β-HSD2 inhibitors in colorectal cancer. Selective inhibition of 11β-HSD2 led to inhibition of the glucocorticoid-dependent COX-2 pathway [24]. Similar observations were made by Yang et al. proving inhibition of the COX-2 pathway and inhibition of 11β-HSD2 activity. The inhibition of 11β-HSD2 represents a modern approach in chemoprevention by increasing the activity of intracellular glucocorticoids in malignant cells. On the basis of the conducted research, the researchers concluded that the inhibition of 11β-HSD2 may be a therapeutic target in chemoprevention as well as an adjuvant to the treatment of colorectal cancer [25]. However, in the studies of Terao et al. different expression of both isoforms of the tested enzyme was demonstrated in skin cells subjected to neoplastic proliferation. 11βHSD1 expression was decreased in contrast to 11β-HSD2 expression. The above reports suggest that the influence of 11β-HSD isoform expression may be a useful diagnostic tool in skin neoplasms [26,27].

### 2.2. The Analysis of the Bioavailability of 2-Tert-Butylaminothiazol-4(5H)-One Derivatives Based on Physicochemical Parameters

The analysis of bioavailability parameters calculated using in silico methods cannot accurately predict the actual bioavailability of newly synthesized compounds; however, the evaluation of these parameters in terms of meeting such rules as Lipinski’s, Veber’s, Ghose’s, or Egan’s rule allows for the selection of compounds that will realistically be good drugs after oral administration [28,29,30,31]. The physicochemical parameters of 2-*tert*-butylaminothiazol-4(5*H*)-one derivatives were determined using Molinspiration [32] and SwissADME [33] programs and are presented in Table 1. 

Table 2 compares the physicochemical parameters of 2-*tert*-butylaminothiazol-4(5*H*)-one derivatives with the criteria of the most important literature rules used to evaluate the bioavailability of drugs. None of the compounds from the obtained series of pseudothiohydantoin derivatives showed deviations from the presented rules, which indicates the probable good bioavailability of these compounds after oral administration.

The logP parameter indicating the lipophilicity of compounds is an important descriptor to be taken into account when evaluating a given compound for inhibition of cytochrome P450 family enzymes [34]. The CYP family enzymes are the main enzymes responsible for drug metabolism in the human body [35]. Inhibition of the metabolism of a drug mediated by an enzyme from the cytochrome P450 group by another drug may lead to increased concentration of this drug in blood and thus to an enhanced therapeutic effect [35]. A study by Gleeson showed that for compounds with logP < 3, the mean IC_50_ values were more than 10 µM indicating that the IC_50_ is above the general literature threshold above which drug–drug interactions are unlikely to be a problem [34]. Among a number of 2-aminothiazol-4(5*H*)-one derivatives, only compound **3g** has a logP > 3, so inhibition of cytochrome P450 enzymes is likely to occur after its use.

## 3. Materials and Methods

### 3.1. General Informations

^1^H- and ^13^C-NMR spectra were recorded on the Bruker Avance 400 and 700 apparatus (TMS as an internal standard). 

HRMS (high resolution mass spectrometry) measurements were made using a Synapt G2 Si mass spectrometer (Waters) equipped with an ESI source and a quadrupole time-of-flight mass analyzer. In order to achieve the highest accuracy of mass measurement, data was collected in center of gravity mode and the mass was corrected during the acquisition using enkephalin leucine solution as external reference (Lock-SprayTM) which generated reference ion at m/z 556.2771 Da ([M + H] ^+^) in positive ESI mode. The measurement results were processed with MassLynx 4.1 software (Waters).

### 3.2. Reagents and Solvents

*Solvents:* chloroform, diethyl ether, dimethylsulfoxide, ethyl acetate, ethyl alcohol, hexane, methyl alcohol-POCh, Poland (Avantor Performance Materials Poland S.A., Gliwice, Poland). 

*Reagents for synthesis: N*-(tert-Butyl)thiourea 97% Alfa Aesar, Kendel Germany, 2-bromo esters: ethyl 2-bromopropionate 99%, 2-bromobutyrate 98%, 2-bromovalerate 99%, 2-bromo-3-methylbutyrate 95%, 2-bromoisobutyrate 98%, 2-bromophenyl acetate 97%, 2-bromo(4-bromophenyl) acetate 97%, bromocyclobutane carboxylate 95% and methyl 1-bromocyclohexane carboxylate 97%-Alfa aesar Kendal Germany, Acros Organic Geel Belgium, Sigma-Aldrich Poznań Poland.

*Auxiliary reagents: N*-ethyldiisopropylamine 99% Alfa Aesar Kendel Germany, hydrochloric acid, magnesium sulfate, sodium and sodium hydroxide-POCh Poland (Avantor Performance Materials Poland S.A., Gliwice, Poland).

*TLC and column chromatography:* 5 × 10 cm TLC plates coated of silica gel with F-254 Merck Darmstadt Germany.

*Column chromatography:* silica gel MN kieselgel 60M with 0.04–0.063 mm grain diameter from Macherey-Nagel, Oensingen, Switzerland.

*11β-HSD1 assays:* carbenoxolone (sodium salt)-Cayman Ann Arbor, MI, USA Chemical Company, cortisone, NADPH tetrasodium salt, phosphate buffer powder, Sigma-Aldrich Poznań Poland, Pooled human liver microsomes, mixed gender, 1 mL, 20 mg/mL Lot No. 1410013-XenoTech, Cortisol Elisa Ref DkO001 Lot No. 4715A-DiaMetra, Spello, Italy), ELISA Kit for 11-Beta-Hydroxysteroid Dehydrogenase Type 1 Lot No.L160706125-Cloud-Clone Corp. Wuhan, China, PBS Lot No. H161008-Pan Biotech Aidenbach, Germany.

*11β-HSD2 assays:* 18-beta-glycyrrhetinic acid-Acros Organic, cortisone, NAD cofactor, phosphate buffer powder-Sigma-Aldrich Poznań Poland, Human Kidney Microsomes, mixed gender, 0.5 mL, 10 mg/mL Lot No. 1710160 XenoTech, Cortisol Elisa Ref DkO001 Lot No. 4715A-DiaMetra Spello Italy, Enzyme-Linked Immunosorbent Assay (ELISA) Kit for 11-Beta-Hydroxysteroid Dehydrogenase Type 2 Lot No. L191113457-Cloud-Clone Corp. Wuhan, China, PBS Lot No. H161008-Pan Biotech Aidenbach, Germany.

### 3.3. Synthesis of Compound 3a, 3f–3g–General Procedure

0.01 mol of *N*-(*tert*-butyl)thiourea (**1**) and 0.011 mol of the corresponding 2-bromoester (**2a**, **2f–2g**) were dissolved in 50 mL of chloroform. The mixture was stirred at room temperature for 4–14 days (TLC control-AcOEt:hexane 1:1). The precipitated crude product was filtered off and crystallized from ethanol [20].

2-(*tert*-Butylamino)-5-methylthiazol-4(5*H*)-one (**3a**)–Yield: 43%. M.p. 235–245 °C. ^1^H-NMR (700 MHz, CDCl_3_, δ ppm, J Hz): 11.93 (s, 1H, NH), 4.36 (q, 1H, C^5^-H, 7.7), 1.76 (d, 3H, C^5^-CH_3_ 7.7), 1.58 (s, 9H, C(CH_3_)_3_). ^13^C-NMR (176 MHz, CDCl_3_, δ ppm): 171.08 (C-4), 171.01 (C-2), 57.85 (C(CH_3_)_3_), 44.57 (C-5), 27.88 (C(CH_3_)_3_), 17.40 (C^5^-CH_3_). HR-MS m/z 187.0907 [M^+^ + 1] (calcd for C_8_H_15_N_2_OS: 187.0905). R_f_ (silicagel, AcOEt:hexane 1:1): 0.33.

2-(*tert*-Butylamino)-5-phenylthiazol-4(5*H*)-one (**3f**)–Yield: 79%. M.p. 222–227 °C. ^1^H-NMR (700 MHz, CDCl_3_, *δ* ppm, J Hz): 11.90 (s, 1H, NH), 7.41–7.44 (m, 3H, C_6_H_5_), 7.35-7.37 (m, 2H, C_6_H_5_), 5.58 (s, 1H, C^5^-H), 1.60 (s, 9H, C(CH_3_)_3_). ^13^C-NMR (176 MHz, CDCl_3_, *δ* ppm): 171.16 (C-4), 169.30 (C-2), 130.95 (1C, C_6_H_5_), 129.71 (1C, C_6_H_5_), 129.32 (2C, C_6_H_5_), 128.34 (2C, C_6_H_5_), 58.01 (C(CH_3_)_3_), 53.63 (C-5), 27.91 (C(CH_3_)_3_). HR-MS *m/z* 249.1071 [M^+^ + 1] (calcd for C_13_H_17_N_2_OS: 249.1062). R_f_ (silicagel, AcOEt:hexane 1:1): 0.45.

5-(4-Bromophenyl)-2-(*tert*-butylamino)thiazol-4(5*H*)-one (**3g**)–Yield: 78%. M.p. 206–207 °C. ^1^H-NMR (700 MHz, CDCl_3_, *δ* ppm, J Hz): 7.56_A_ (d, 2H, C_6_H_4_, 8.4), 7.45_C_ (d, 2H, C_6_H_4_, 8.4), 7.23_A_ (d, 2H, C_6_H_4_, 8.4), 7.20_C_ (d, 2H, C_6_H_4_, 8.4), 6.10_A_ (s, 1H, NH), 5.50_C_ (s, 1H, OH), 5.47_A_ (s, 1H, C^5^-H), 5.16_C_ (s, 1H, C^5^-H), 1.59_A_ (s, 9H, C(CH_3_)_3_), 1.52_C_ (s, 9H, C(CH_3_)_3_). ^13^C-NMR (176 MHz, CDCl_3_, *δ* ppm): 187.72 (C-4), 177.28 (C-2), 134.91_A_ (1C, C_6_H_4_), 132.42_A_ (2C, C_6_H_4_), 131.62_C_ (2C, C_6_H_4_), 130.59_C_ (1C, C_6_H_4_), 129.86_A_ (2C, C_6_H_4_), 129.64_C_ (2C, C_6_H_4_), 123.86_C_ (1C, C_6_H_4_), 121.89_A_ (1C, C_6_H_4_), 58.31_A_ (C(CH_3_)_3_), 57.44_C_ (C(CH_3_)_3_), 56.42_C_ (C-5), 53.80_A_ (C-5), 28.41_C_ (C(CH_3_)_3_), 28.10_A_ (C(CH_3_)_3_). HR-MS *m/z* 327.0174 [M^+^ + 1] (calcd for C_13_H_16_^79^BrN_2_OS: 327.0167). R_f_ (silicagel, AcOEt:hexane 1:1): 0.47.

### 3.4. Synthesis of Compound 3b–3e–General Procedure

30 mL of methyl alcohol were placed in the flask and 0.46 g (0.02 mol) of sodium was added. Then 0.59 g (0.01 mol) of *N*-(*tert*-butyl)thiourea (**1**) and 0.011 mol of the corresponding 2-bromoester (**2b**–**2e**) were added. The mixture was heated for 10-18 h (TLC control-AcOEt:hexane 1:1). The solvent was evaporated on a rotary evaporator and the residue was dissolved in 20 mL of water and neutralized with 2M HCl to pH = 7–8. The crude product was extracted with chloroform. The organic layer was dried with MgSO_4_, filtered and after solvent evaporated crystallized from diethyl ether [36].

2-(*tert*-Butylamino)-5-ethylthiazol-4(5*H*)-one (**3b**)–Yield: 34%. M.p. 152–154 °C. ^1^H-NMR (700 MHz, CDCl_3_, *δ* ppm, J Hz): 6.1 (s, 1H, NH), 4.14 (dd, 1H, C^5^-H 4.2 8.4), 2.13–2.18 (m, 1H, C^5^-CH_A_-CH_3_), 1.84–1.92 (m, 1H, C^5^-CH_B_-CH_3_), 1.47 (s, 9 H, C(CH_3_)_3_), 0.99 (t, 3H, CH_2_-CH_3_, 7.7). ^13^C-NMR (176 MHz, CDCl_3_, *δ* ppm): 190.43 (C-4), 177.91 (C-2), 57.81 (C(CH_3_)_3_), 55.91 (C-5) 28.40 (C(CH_3_)_3_), 26.10 (C^5^-CH_2_CH_3_), 11.40 (C^5^-CH_2_CH_3_). HR-MS *m/z* 201.1059 [M^+^ + 1] (calcd for C_9_H_17_N_2_OS: 201.1062). R_f_ (silicagel, AcOEt:hexane 1:1): 0.40.

2-(*tert*-Butylamino)-5-propylthiazol-4(5*H*)-one (**3c**)–Yield: 37%. M.p. 131–132 °C. ^1^H-NMR (700 MHz, CDCl_3_, *δ* ppm, J Hz): 5.66 (s, 1H, NH), 4.17 (dd, 1H, C^5^-H 4.2 9.1), 2.15–2.19 (m, 1H, C^5^-CH_A_CH_2_CH_3_), 1.75-1.79 (m, 1H, C^5^-CH_B_CH_2_CH_3_), 1.48 (s, 9 H, C(CH_3_)_3_), 1.39–1.47 (m, 2H, C^5^-CH_2_CH_2_-CH_3_), 0.95 (t, 3H, C^5^-CH_2_CH_2_CH_3_, 7.0). ^13^C-NMR (176 MHz, CDCl_3_, *δ* ppm): 190.61 (C-4), 177.84 (C-2), 56.40 (C(CH_3_)_3_), 55.97 (C-5), 35.19 (C^5^-CH_2_CH_2_CH_3_), 28.43 (C(CH_3_)_3_), 21.16 (C^5^-CH_2_CH_2_CH_3_), 13.19 (C^5^-CH_2_CH_2_CH_3_). HR-MS *m/z* 215.1219 [M^+^ + 1] (calcd for C_10_H_19_N_2_OS: 215.1218). R_f_ (silicagel, AcOEt:hexane 1:1): 0.48.

2-(*tert*-Butylamino)-5-(propan-2-yl)thiazol-4(5*H*)-one (**3d**)–Yield: 27%. M.p. 175–177 °C. ^1^H-NMR (700 MHz, CDCl_3_, *δ* ppm, J Hz): 5.83 (s, 1H, NH), 4.25 (d, 1H, C^5^-H 2.8), 2.55–2.60 (m, 1H, C^5^-CH), 1.47 (s, 9H, C(CH_3_)_3_), 1.03 (d, 3H, CH-CH_3A_, 7.0), 0.99 (d, 3H, CH-CH_3B_, 7.0). ^13^C-NMR (176 MHz, CDCl_3_, *δ* ppm): 190.28 (C-4), 178.29 (C-2), 63.65 (C(CH_3_)_3_), 55.79 (C-5), 30.30 (C^5^-CH), 28.35 (C(CH_3_)_3_), 22.09 (CH(CH_3A_), 15.74 (CH(CH_3B_). HR-MS *m/z* 215.1222 [M^+^ + 1] (calcd for C_10_H_19_N_2_OS: 215.1218). R_f_ (silicagel, AcOEt:hexane 1:1): 0.46. 

2-(*tert*-Butylamino)-5,5-dimethylthiazol-4(5*H*)-one (**3e**)–Yield: 40%. M.p. 212–217 °C. ^1^H-NMR (700 MHz, CDCl_3_, *δ* ppm, J Hz): 5.61 (s, 1H, NH), 1.64 (s, 6H, C^5^(CH_3_)_2_), 1.49 (s, 9H, C(CH_3_)_3_). ^13^C-NMR (176 MHz, CDCl_3_, *δ* ppm): 193.38 (C-4), 176.12 (C-2), 60.65 (C(CH_3_)_3_), 55.79 (C-5), 28.46 (C(CH_3_)_3_), 27.59 (2C, C^5^-(CH_3_)_2_). HR-MS *m/z* 201.1064 [M^+^ + 1] (calcd for C_9_H_17_N_2_OS: 201.1062). R_f_ (silicagel, AcOEt:hexane 1:1): 0.44.

### 3.5. Synthesis of Compound 3h–3i–General Procedure

2 mL of *N*,*N*-diisopropylethylamine, 0.01 mol of *N*-(*tert*-butyl)thiourea (**1**) and 0.01 mol of the 2-bromoester (**2h**-**2i**) were dissolved in 3.5 mL of ethyl alcohol. It was heated to reflux for 7-14 days (TLC control-AcOEt:hexane 1:1). After the solvent was evaporated, the residue was dissolved in 10 mL of water. The product was extracted with chloroform. The organic layer was dried with anhydrous MgSO_4_. After filtered off and evaporated of the solvent the reaction mixture was purified by column chromatography [20].

2-(*tert*-Butylamino)-1-thia-3-azaspiro[4.5]dec-2-en-4-one (**3h**)–Yield: 12%. M.p. 218–220 °C. ^1^H-NMR (700 MHz, CDCl_3_, δ ppm, J Hz): 5.57 (s, 1H, NH), 2.08–2.12 (m, 2H, C_5_H_10_), 1.90–1.93 (m, 2H, C_5_H_10_), 1.82–1.87 (m, 2H, C_5_H_10_), 1.72–1.74 (m, 1H, C_5_H_10_), 1.49 (s, 9H, C(CH_3_)_3_), 1.27–1.40 (m, 3H, C_5_H_10_). ^13^C-NMR (176 MHz, CDCl_3_, δ ppm): 192.91 (C-4), 177.13 (C-2), 70.21 (C-5), 55.77 (C(CH_3_)_3_, 36.34 (2C, C_5_H_10_), 28.84 (C(CH_3_)_3_), 25.07 (2C, C_5_H_10_), 24.47 (1C, C_5_H_10_). HR-MS m/z 241.1376 [M^+^ + 1] (calcd for C_12_H_21_N_2_OS: 241.1375). R_f_ (silicagel, AcOEt:hexane 1:1): 0.5.

6-(*tert*-Butylamino)-5-thia-7-azaspiro[3.4]oct-6-en-8-one (**3i**)–Yield: 16%. M.p. 211–213 °C. ^1^H-NMR (700 MHz, CDCl_3_, δ ppm, J Hz): 5.78 (s, 1H, NH), 2.78–2.86 (m, 2H, C_3_H_6_), 2.46–2.50 (m, 2H, C_3_H_6_), 2.26–2.30 (m, 1H, C_3_H_6_), 1.98–2.04 (m, 1H, C_3_H_6_), 1.47 (s, 9H, C(CH_3_)_3_). ^13^C-NMR (176 MHz, CDCl_3_, δ ppm): 193.46 (C-8), 176.50 (C-6), 62.01 (C-4), 56.17 (C(CH_3_)_3_), 34.28 (2C, C_3_H_6_), 28.87 (C(CH_3_)_3_), 16.93 (1C, C_3_H_6_). HR-MS m/z 213.1062 [M^+^ + 1] (calcd for C_10_H_17_N_2_OS: 213.1062). R_f_ (silicagel, AcOEt:hexane 1:1): 0.5.

### 3.6. Inhibition of 11β-HSD1 Assays

The transformation of cortisone to cortisol was performed in 96-well microtiter plates using human liver microsomes as a source of 11β-HSD1 in a total volume of 100 µL. Then, 20 μL of cortisone/NADPH (final concentration 200 nM/2 μM), 10 μL of microsomes (1.13 μg/mL 11β-HSD1) in PBS (final amount of 2.5 μg), 60 μL of phosphate buffer (pH 7.4) and 10 µL of inhibitor solution (DMSO solvent/water 1/99, 10 µM final concentration) was placed in the well. The resulting solution was incubated for 150 min at 37 °C. The addition of 10 µL of a 100 µM solution of 18β-glycyrrhetinic acid in PBS stopped the reaction [37]. Measurement of the obtained amount of cortisol was performed with a cortisol ELISA kit.

### 3.7. Inhibition of 11β-HSD2 Assays

Transformation of cortisol to cortisone was performed on 96-well microtiter plates using human kidney microsomes as a source of 11*β*-HSD2 in a total volume of 100 μL. Then, 20 μL of substrate mixture cortisol/NAD^+^ (final concentration 200 nM/2 μM), 10 μL of microsomes (0.127 μg/mL 11*β*-HSD2) solution in PBS (final quantity 2.5 μg), 60 μL of phosphate buffer (pH 7.4) and 10 μL of inhibitor solution (solvent DMSO/water 1/99, final concentration 10 μM) were mixed in the well. The resulting solutions were incubated for 150 min at 37 °C. The addition of 10 μL solution containing 100 μM carbenoxolone in PBS stopped the reaction [37]. The amount of unreacted cortisol was measured using a cortisol ELISA kit.

### 3.8. Determination of IC_50_

IC_50_ values were obtained by performing similar assay as described in Section 3.6 (for inhibitors of 11β-HSD1) or at Section 3.7 (for inhibitors of 11β-HSD2) with inhibitors at concentrations of 10, 5, 2.5, 1.25, 0.625 µM. The next step was to plot the dependence of cortisol concentration (in the case of 11β-HSD1 inhibitors) and cortisone (in the case of 11β-HSD2 inhibitors) obtained in response to the concentration of the inhibitor. The inhibitor concentration that resulted in a 50% reduction in cortisol (or cortisone) compared to the blank (no inhibitor reaction) was read from the graph.

## 4. Conclusions

In conclusion, we obtained the nine novel *N*-*tert*-butyl substituted 2-aminothiazol-4(5*H*)-one (pseudothiohydantoin) derivatives, differing in the substituents at C-5 of the thiazole ring. The inhibitory activity and selectivity of the obtained derivatives in relation to two isoforms of 11β-hydroxysteroid dehydrogenase (type 1 and type 2) were evaluated. The **3h** derivative, containing the cyclohexane substituent at the 5-position of the thiazole ring in the spiro system which inhibits the enzyme isoform 1 activity by 82.5% at a concentration of 10 µM, has the highest inhibitory activity in relation to 11β-HSD1. The derivative with the phenyl substituent at C-5 showed the highest inhibition of 11β-HSD2 (53.57% at a concentration of 10 µM). A low selectivity in the inhibition of 11β-HSD2 was observed, but unlike 18β-glycyrrhetinic acid these compounds inhibit the activity of 11β-HSD2 to a greater extent than that of 11β-HSD1.

The obtained results may constitute an innovative approach in cancer therapy, aimed especially at the action of 11βHSD1 and 11βHSD2. It may have a significant impact on the correct response and action of glucocorticoids, which in turn may be of key importance in inhibiting the proliferation of neoplastic cells.

## Figures and Tables

**Table 1:** Physicochemical properties calculated for compounds **3a**–**3i** using Molinspiration and SwissADME software.

Compound	Molecular Weight ^a^ [g/mol]	HBD ^a^	HBA ^a^	Topological Polar Surface Area ^a^ [A^2^]	Number Rotatable Bonds ^a^	Molar Refractivity ^b^	miLogP ^a^	LogP ^b^
**3a**	186.28	1	3	41.46	2	56.10	1.26	1.50
**3b**	200.31	1	3	41.46	3	60.90	1.77	1.87
**3c**	214.33	1	3	41.46	4	65.71	2.33	2.21
**3d**	214.33	1	3	41.46	3	65.71	2.01	2.14
**3e**	200.31	1	3	41.46	2	60.94	1.71	1.83
**3f**	200.48	1	3	41.46	3	75.78	2.48	2.53
**3g**	327.25	1	3	41.46	3	83.48	3.29	3.16
**3h**	240.37	1	3	41.46	2	73.25	2.88	2.62
**3i**	212.32	1	3	41.46	2	63.64	1.63	2.00

Abbreviations: HBD-hydrogen bond donor; HBA-hydrogen bond acceptor; Molininspiration ^a^; SwissADME ^b^.

**Table 2:** Summary of the most important literature criteria considered in the assessment of bioavailability of new compounds.

Rule	Criteria	3a	3b	3c	3d	3e	3f	3g	3h	3i
		(Yes/No)								
**Lipinski**	**MW ≤ 500 Da** **LogP ≤ 5** **HBD ≤ 5** **HBA ≤ 10**	**Yes**	**Yes**	**Yes**	**Yes**	**Yes**	**Yes**	**Yes**	Yes	Yes
Veber	tPSA ≤ 140 A^2^ Nrotb ≤ 10	Yes	Yes	Yes	Yes	Yes	Yes	Yes	Yes	Yes
Egan	0 ≥ tPSA ≤ 132 A^2^ −1 ≥ logP ≤ 6	Yes	Yes	Yes	Yes	Yes	Yes	Yes	Yes	Yes
Ghose	160 ≤ MW ≤ 480 −0.4 ≤ logP ≤ 5.6 40 ≤ MR ≤ 130 20 ≤ atoms ≤ 70	Yes	Yes	Yes	Yes	Yes	Yes	Yes	Yes	Yes

Abbreviations: MW—molar mass; HBD—hydrogen bond donor; HBA—hydrogen bond acceptor; tPSA—topological polar surface area; Nrotb—number of rotatable bonds; MR—molar refraction.

## Data Availability

Data available from the authors.

## Sample Availability

Samples of the compounds **3a**–**3i** are available from the authors.
